# Longitudinal Trends of Salivary Oxidized Thymosin β4 and β10 in Preterm Infants with Bronchopulmonary Dysplasia

**DOI:** 10.3390/children13060770

**Published:** 2026-06-02

**Authors:** Chiara Tirone, Simona Fattore, Nicoletta Menzella, Davide De Tomaso, Martina Giaimo, Stefano Cecere, Alessandro Perri, Irene Messana, Tiziana Cabras, Barbara Manconi, Alessandra Olianas, Cristina Contini, Giulia Guadalupi, Gavino Faa, Massimo Castagnola, Federica Iavarone, Giovanni Vento

**Affiliations:** 1 Neonatology Unit, Department of Women and Child Health, Fondazione Policlinico Universitario Agostino Gemelli IRCCS, 00168 Rome, Italy; chiara.tirone@policlinicogemelli.it (C.T.);; 2 Istituto di Scienze e Tecnologie Chimiche “Giulio Natta”, Consiglio Nazionale delle Ricerche, 20132 Milan, Italy; 3 Dipartimento di Scienze della Vita e dell’Ambiente, Sezione Biomedica, Università di Cagliari, 09042 Monserrato, Italy; 4 Dipartimento di Scienze Mediche e Sanità Pubblica, Università di Cagliari, 09042 Monserrato, Italy; 5 Department of Biology, College of Science and Technology, Temple University, Philadelphia, PA 19122, USA; 6 Laboratorio di Proteomica, Centro Europeo di Ricerca sul Cervello, Fondazione Santa Lucia IRCCS, 00179 Rome, Italy; 7 Clinical Chemistry, Biochemistry and Molecular Biology Operations (UOC), Fondazione Policlinico Universitario Agostino Gemelli IRCCS, 00168 Rome, Italy; 8 Dipartimento di Scienze Biotecnologiche di Base, Cliniche Intensivologiche e Perioperatorie, Facoltà di Medicina e Chirurgia, Università Cattolica Sacro Cuore, 00168 Rome, Italy; 9 Divisione di Neonatologia, Dipartimento per la Salute della Donna e del Bambino, Università Cattolica del Sacro Cuore, 00168 Rome, Italy

**Keywords:** proteomics, preterm newborns, saliva proteome, thymosin, oxidized Tβ4, oxidized Tβ10, bronchopulmonary dysplasia

## Abstract

**Highlights:**

**What are the main findings?**

- No significant differences in salivary thymosin β4 and β10 were observed during the first week of life between neonates who later developed BPD and those who did not.
- Longitudinal trajectories of thymosin β4 and β10 diverged between neonates with and without BPD.

**What are the implications of the main findings?**

- Longitudinal trajectories of Tβ4 and Tβ10 differed between infants with and without BPD. Postnatal changes in these proteins may be associated with differences in clinical course and exposure to postnatal oxidative stress. Oxidized Tβ10 forms represent a promising tool for exploring oxidative status in preterm neonates.
- This study highlights how salivary proteomic analysis is a non-invasive approach that is well tolerated even by extremely preterm neonates, enabling longitudinal monitoring.

**Abstract:**

Background: Oxidative stress plays a key role in the pathogenesis of complications in preterm infants, including bronchopulmonary dysplasia (BPD). Thymosin β4 (Tβ4) and thymosin β10 (Tβ10) are proteins involved in tissue repair and responses to oxidative stress, but their role in extremely preterm neonates remains poorly understood. Methods: A total of 149 saliva samples from 18 infants with gestational age < 30 weeks were analyzed. Salivary proteins and their proteoforms were characterized using an integrated proteomic platform based on nano-HPLC-ESI-MS. Relative quantification was performed using extracted ion current (XIC) peak areas. Associations with postmenstrual age, oxygen requirement, and BPD development were assessed, including longitudinal analysis using generalized estimating equation (GEE) models. Results: Significant correlations were found between postmenstrual age and total Tβ4 (*p* = 0.001), oxidized Tβ4 percentage (*p* = 0.025), and total Tβ10 (*p* = 0.043). Higher oxygen requirement was associated with lower levels and percentages of oxidized Tβ10 (*p* = 0.005; *p* < 0.001). No significant differences were observed during the first week of life between neonates who later developed BPD and those who did not. However, longitudinal analysis showed that in neonates without BPD, total and oxidized Tβ10 and total Tβ4 increased over time, whereas in neonates with BPD, these biomarkers remained stable or decreased. The increase in oxidized Tβ10 percentage was slower in infants with BPD. Conclusions: Although no early differences were detected, longitudinal trajectories of Tβ4 and Tβ10 differed between infants with and without BPD. Postnatal changes in these proteins may be associated with differences in clinical course and exposure to postnatal oxidative stress.

## 1. Introduction

Since intrauterine life, there is a delicate balance between the production of reactive oxygen species (ROS) and the body’s antioxidant defense mechanisms. When this situation becomes unbalanced and free radical production exceeds the capacity of antioxidant systems, a condition referred to as “oxidative stress” occurs [1]. When produced in limited amounts, ROS have a beneficial effect, stimulating cell regeneration and immune function. At high concentrations, however, they result in an imbalance that can damage DNA, lipids, and proteins. Irreversible oxidative protein damage results from covalent modifications, including carbonylation of arginine, lysine, proline, and threonine, and oxidation of thiols, which can result in misfolding and fragmentation [2,3].

Neonates, especially those born preterm (before 37 weeks of gestational age), have underdeveloped antioxidant systems, making them particularly susceptible to oxidative stress. Maternal conditions such as diabetes, obesity, and pre-eclampsia, which are more prevalent in preterm births, can worsen the situation by exposing the fetus to increased oxidative stress [4,5]. A growing body of evidence consistently indicates that maternal thyroid dysfunction—such as overt hypothyroidism and hyperthyroidism—during pregnancy is associated with a higher risk of adverse obstetric and neonatal outcomes, including preeclampsia, gestational diabetes, placental abruption, preterm birth, fetal death, and low birth weight. In particular, hyperthyroidism during pregnancy has an oxidative stress-promoting effect, leading to potential cellular damage [6,7,8]. Furthermore, intrauterine growth restriction and macrosomia appear to be associated with mitochondrial dysfunction and subsequent increased production of ROS [9]. In preterm infants, the increased risk of resuscitation at delivery compared to term infants, associated with episodes of apnea, infections, and longer duration of mechanical ventilation and oxygen therapy in the Neonatal Intensive Care Unit (NICU), contribute to a higher incidence of oxidative stress-related diseases such as bronchopulmonary dysplasia (BPD), retinopathy of prematurity (ROP), intraventricular hemorrhage (IVH), periventricular leukomalacia (PVL), and necrotizing enterocolitis (NEC) [10,11,12]. The mechanisms by which oxidative stress contributes to the development of “oxidative stress related pathology” are only partially known. In PVL, increased ROS levels during reperfusion after hypoxic events are implicated in apoptosis of oligodendrocyte precursors [13,14]. In NEC, free radicals produced via inflammation, ischemia, and reperfusion contribute to the disruption of the immature gut barrier [15,16].

BPD is the most common morbidity in surviving preterm infants, especially those born before 28 weeks of gestation. Among the known risk factors (prenatal and postnatal) that increase the incidence of this disease, the need for oxygen therapy and oxidative stress play a key role [17]. In immature lungs, mechanical ventilation and oxygen administration result in activation of inflammatory cytokines, which in turn lead to ROS production through activation of neutrophil degranulation. This condition, associated with the immaturity of antioxidant systems, results in the predominance of oxidative stress [18,19]. Several studies have shown that premature infants with BPD, already in the early stages of the disease, exhibit a different oxidation pattern compared to infants who will not develop BPD [20,21,22]. Most of these studies have focused on plasma and bronchoalveolar lavage fluid (BALF) analyses [23,24,25,26,27,28]. Notably, infants who developed BPD showed significantly higher protein oxidation in tracheal aspirates, regardless of gestational age [23,24]. Oxidative inactivation of alpha-1-antitrypsin, measured in BALF samples from preterm infants, was found to correlate with the development of BPD [21]. Increased plasma levels of heptanal, 2-nonenal, and 4-hydroxynonenal were found to be good predictors of BPD [25]. Additionally, lipid peroxidation, measured in cord blood, is significantly associated with duration of oxygen treatment and respiratory support [26]. Other promising markers are currently under investigation [28,29].

**Figure 1:**
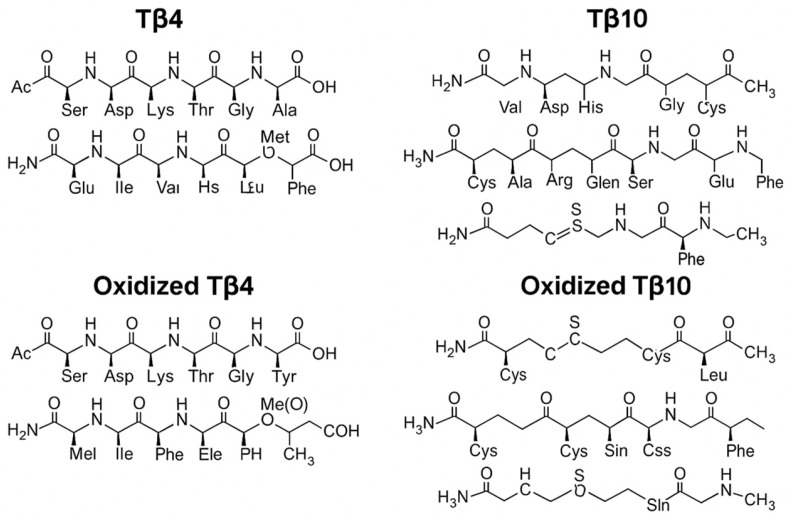
Full amino acid sequences shown as peptide chain structures of Tβ4 and Tβ10 and oxidized Tβ4 and Tβ10.

Thymosins are a family of small proteins widely distributed across tissues and involved in numerous physiological and pathological processes, including cytoskeletal reorganization, cell migration, immune response modulation, and tissue repair. Among them, thymosins β comprise approximately 20 polypeptides, with a molecular weight of about 5 kDa, comprising 40–44 amino acid residues. It appears that mammals express mainly thymosin β4 (Tβ4) and, in humans, also thymosin β10 (Tβ10) [30]. Tβ4 and Tβ10 are the most extensively studied proteoforms in the context of cellular stress and inflammation, and both contain a methionine residue prone to oxidation at position 6 of the amino acid sequence [31,32]. Figure 1 shows the chemical structure of oxidized/non-oxidized Tβ4 and Tβ10. In particular, Tβ4 is known for its role in promoting angiogenesis, protecting against oxidative damage, and supporting tissue regeneration [33]. Recent evidence suggests that the selective oxidation of thymosins, leading to sulfoxidized derivatives at methionine residues, does not merely represent molecular damage but rather a functionally relevant post-translational modification (PTM). Oxidized forms of Tβ4 appear to exert cytoprotective and anti-inflammatory actions, contributing to the regulation of the oxidative stress response with a predominantly inhibitory action on damage induced by oxidative stress itself. The data show that Tβ4-sulfoxide (Tβ4-SO) is formed through oxidation of Tβ4 in inflamed tissues where neutrophil oxidative bursts occur [34]. This suggests its role as a local feedback regulator that both limits inflammation and oxidative damage [34,35]. Young et al. in 2001 showed that Tβ4-SO suppresses neutrophil chemotaxis and oxidative burst, increases neutrophil apoptosis, and promotes their clearance by macrophages [35]. This molecule exhibits a reduced affinity for monomeric actin, yet it retains significant biological activity [36]. It is believed that Tβ4-SO plays a role in modulating immune cells, inhibiting interferon-γ activity, and positively contributing to wound healing and other inflammation-related processes [34]. Moreover, experimental studies have demonstrated, for instance, that oxidized Tβ4 can modulate inflammatory responses in epithelial and cardiac cells; Tβ4 has a protective role against lung tissue damage, while its oxidation product mirrors an alveolar inflammatory status, promoting mechanisms of damage resolution [34,37,38]. There are no specific studies on the oxidation of Tβ10 and its biological role, comparable to those clearly documented for Tβ4-SO. What is known is that Tβ10 appears to be involved in cytoskeletal regulation, cellular development and differentiation; moreover, it seems to play an important role in oncological pathology [31,32,39,40,41].

In preterm neonates, whose early exposure to pro-oxidant conditions may contribute to the development of chronic inflammatory diseases such as BPD, the analysis of oxidized thymosins may offer insight into individual variability in oxidative stress responses. In this context, saliva emerges as an ideal biological fluid for proteomic studies in neonates: it is non-invasive, easily collectable at multiple time points, and allows for the investigation of PTMs such as oxidation [42].

Therefore, the characterization of oxidized derivatives of Tβ4 and Tβ10 and determination of their levels in the saliva of preterm infants may offer new insights for the identification of possible early oxidative stress biomarkers and for a better understanding of the pathophysiological mechanisms underlying BPD.

The aim of this study was to investigate the relative amounts of oxidized Tβ4 and Tβ10 in the oral cavity of preterm infants at birth and later stages of postnatal life through nano-High-Performance Liquid Chromatography (HPLC)–Electrospray Ionization (ESI)–Mass Spectrometry (MS) (nano-HPLC-ESI-MS) analysis of oral fluid.

Considering the evidence supporting a protective role of Tβ4 against oxidative damage in other pathological conditions, we aimed to evaluate a possible association between oxidized Tβ4 levels and the increased risk and severity of preterm-related diseases such as BPD, in which oxidative stress is recognized as a key pathogenic factor. We extended the analysis to include oxidized Tβ10 as well.

## 2. Materials and Methods

### 2.1. Setting

The enrollment of newborns and the collection of saliva samples were performed at the Division of Neonatology of the Fondazione Policlinico Universitario A. Gemelli IRCCS of Rome. The treatment and proteomic analysis of the collected saliva samples were performed at the Dipartimento di Scienze biotecnologiche di base, cliniche intensivologiche e perioperatorie of the Università Cattolica del Sacro Cuore of Rome.

The Unità di Proteomica e Metabolomica of IRCCS-Fondazione Santa Lucia of Roma, the Dipartimento di Scienze della Vita e dell’Ambiente and Sezione di Anatomia Patologica of the Dipartimento di Scienze Mediche e Sanità Pubblica of Cagliari University, and the Istituto di Scienze e Tecnologie Chimiche “Giulio Natta”, Consiglio Nazionale delle Ricerche, contributed to the analysis of saliva samples and to data processing.

### 2.2. Study Population and Inclusion Criteria

The study was carried out in accordance with the ethical standards laid down in the 1964 Declaration of Helsinki. The study was approved by the Ethics Committee of the Fondazione Policlinico Universitario A. Gemelli IRCCS—Università Cattolica del Sacro Cuore on 23 September 2021 (protocol number: 0032898/21, study ID: 4242). All rules were observed, and written consent forms were signed by the parents or legal guardians of each child. For ethical reasons, saliva was collected only when sample collection caused no stress. All parents or legal guardians of the eligible newborns signed the informed consent form and there were no refusals to participate in the study.

The criteria for the study included preterm newborns with gestational age between 175–216 days (25–30 weeks), admitted to the Neonatal Intensive Care Unit (NICU) from January 2024 to January 2025. Infants with major congenital malformations or prenatal infections were excluded from the study.

Saliva samples were collected at least seven days after birth and thereafter every 7–10 days up to 40 weeks (286 days) of Post-Menstrual Age (PMA) or up to discharge if it occurred earlier.

Data on clinical course, including respiratory support, i.e., mechanical ventilation and non-invasive ventilation (NIV), oxygen duration, and postnatal complications such as IVH, sepsis, pneumonia, NEC, and ROP, were collected and recorded for each patient to assess their potential impact on salivary biomarker levels.

As this was a pilot study, in the “pre-discovery” phase of peptide characterization, the calculation of the sample size was not feasible.

However, it was possible to estimate the number of patients who could be enrolled in an appropriate timeframe: considering that in 2023, 50 newborns with gestational age (GA) between 25 and 30 weeks were hospitalized in the NICU, and among these, 13 (26%) died, it was estimated that at least 18 infants could be studied over a period of approximately 12 months. The number of infants we intended to study was in line with the literature evidence for a pilot study [43].

From the 18 enrolled newborns, a total of 149 samples were collected during the period between birth and 40 weeks of PMA, or until discharge if it occurred earlier.

### 2.3. Sample Collection and Treatment

The whole saliva was collected with a soft plastic aspirator as it flowed into the anterior floor of the mouth. After collection, each saliva sample was immediately diluted 1:1 (*v*/*v*) with 0.2% aqueous 2,2,2-trifluoroacetic acid (TFA) in an ice bath. The solution was then centrifuged at 8000× *g* for 5 min (4 °C). Finally, the acidic supernatant was separated from the pellet and either immediately analyzed with the HPLC-ESI-MS apparatus or stored at −80 °C until analysis.

### 2.4. RP–HPLC-ESI-MS Analysis

The protein content of every sample was determined using the Bradford Protein Assay (Bio-Rad Laboratories, Hercules, CA, USA), and the same total protein amount was used for mass spectrometry analysis.

An UltiMate 3000 Rapid Separation Liquid Chromatography (RSLC) nano-HPLC System (Thermo Fisher Scientific, Waltham, MA, USA) coupled with a high-resolution Orbitrap Fusion Lumos Tribrid Mass Spectrometer (Thermo Fisher Scientific, Waltham, MA, USA) equipped with an ESI source was used. Peptides were separated on a PepMap RSLC C18 column (2 µM, 100 Å, 50 µm × 15 cm, Thermo Fisher Scientific, Waltham, MA, USA) using gradient elution. Eluent A consisted of an aqueous solution of 0.1% formic acid (FA), while eluent B was Acetonitrile (ACN) with 0.1% FA. The gradient program was as follows (total runtime: 155 min): 3% B and 97% A (min 0–110), 20% B and 80% A (min 110–120), 40% B and 60% A (min 120–125), 90% B and 10% A (min 125–145), and 3% B and 97% A (min 145–155), with a flow rate of 0.3 μL/min. Each injection volume was 5 μL (containing a total of 1 μg of proteins), with a Nanospray Ionization (NSI) ion source type, positive polarity (voltage 1800 V). MS parameters included data-dependent scan mode (DDS) for acquiring high-resolution MS/MS spectra with an Orbitrap detector, a resolution of 120,000 in the 375–1500 *m*/*z* range, and Higher-energy Collisional Dissociation (HCD) fragmentation. Samples were analyzed in analytical triplicate.

### 2.5. Intact Protein/Peptide Characterization and Relative Quantification

The different salivary proteins and their proteoforms investigated were characterized by means of an integrated proteomic platform.

**Figure 2:**
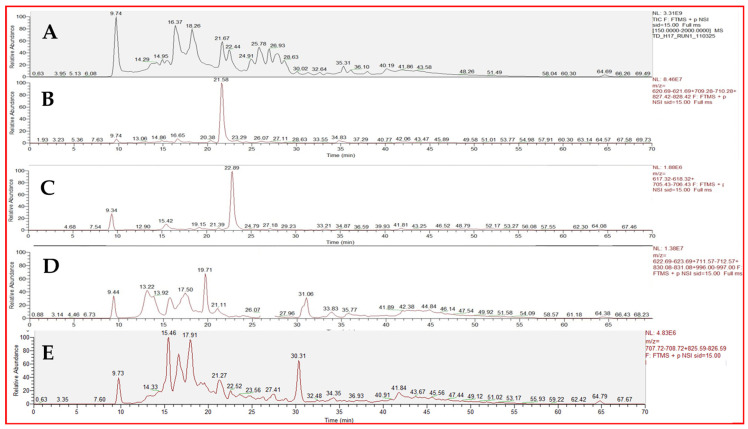
(**A**) Total ion current (TIC) of a salivary profile. (**B**) Extracted ion current (XIC) of Tβ4 RT 21.58. (**C**) XIC of Tβ10 RT 22.89. (**D**) XIC of oxidized Tβ4 RT 19.71. (**E**) XIC oxidized Tβ10 RT 21.27.

Salivary peptide and protein quantitative analysis of the saliva specimens was based on the measurement of the extracted ion current (XIC) peak area (signal/noise ratio > 5). The XIC search revealed the peak associated with the protein of interest by extracting from the total ion current (TIC) chromatographic profile the intensity of the ion current of specific multiply charged ions (*m*/*z*) generated by the ESI source (Figure 2). The ions used to quantify the proteins and peptides were chosen in a number roughly proportional to the protein mass and carefully selected to exclude values in common with other co-eluting peptides. The area of the XIC peak was proportional to protein/peptide concentration; therefore, under constant analytical conditions, it was used for quantitative analysis and comparative studies [44,45,46]. The estimated percentage error of the XIC procedure was <10%.

The percentage of oxidized Tβ4 and Tβ10 was calculated as the percentage of the XIC area of the oxidized forms relative to the XIC area of both total Tβ4 and total Tβ10, given by the sum of the oxidized and non-oxidized forms of the protein.

### 2.6. Statistical Analysis

Statistical analysis was conducted using Statistical Package for Social Science (SPSS^®^, IBM^®^) version 25. Categorical data were expressed as number and percentage and numerical data were reported as mean and standard deviation (SD) or median and interquartile range (IQR), depending on their distribution.

The Shapiro–Wilk test was employed to assess the normality of distribution for continuous variables. Categorical data were compared using the Chi-square test or Fisher’s exact test, as appropriate. Continuous variables were compared using the Student’s *t*-test for normally distributed data or the Mann–Whitney U test for non-normally distributed data. To address the non-normal distribution of biomarker levels, thymosin β4 and β10 concentrations (total and oxidized) were log-transformed using the natural logarithm (ln) prior to analysis. Percentage values were analyzed on the original scale.

Correlations between variables were assessed using Pearson’s correlation coefficient for parametric data or Spearman’s rank correlation coefficient for non-parametric data.

Longitudinal changes in salivary thymosin levels were analyzed using Generalized Estimating Equations (GEE) to account for repeated measurements within subjects. An autoregressive correlation structure (AR-1) was specified, assuming that measurements closer in time are more strongly correlated. In the GEE models, time (week of life), bronchopulmonary dysplasia (BPD), and their interaction (BPD × time) were included as predictors to assess differences in temporal trends between groups. Results are reported as regression coefficients (β) with 95% confidence intervals (95% CI).

A *p*-value < 0.05 was considered statistically significant.

## 3. Results

### 3.1. Patients’ Demographic and Clinical Characteristics

We enrolled 18 preterm infants with a gestational age (GA) of less than 30 weeks. General characteristics of the enrolled neonates are shown in Table 1. Among them, seven infants (38.9%) developed BPD: according to the Jensen definition, two infants had Grade 1 BPD and give infants had Grade 2 BPD [47].

To address the multi-factorial nature of BPD development, we compared the clinical profile of the 7 infants who developed BPD with that of the 11 infants of the Non-BPD group. As shown in Table 2, infants in the BPD group were significantly more premature (GA 25.65 vs. 28.95 weeks, *p* = 0.001) and had lower birth weights (760 g vs. 1130 g, *p* = 0.001). Beyond gestational age, the BPD group exhibited a significantly higher burden of postnatal morbidities. The incidence of severe intraventricular hemorrhage (IVH > grade 2) was 42.9% in the BPD group compared to 0% in controls (*p* = 0.043). Similarly, the BPD group had a significantly higher incidence of culture-proven sepsis (*p* = 0.006) and pneumonia (*p* = 0.003). No cases of necrotizing enterocolitis (NEC) were observed in either group. Infants who developed BPD also showed greater respiratory support needs, including longer duration of mechanical ventilation, non-invasive ventilation, and oxygen therapy.

### 3.2. Samples Collection

A total of 149 saliva samples were analyzed. The time frame between the collection of the saliva samples and the receipt of the analysis results for Tβ4 and Tβ10 was approximately 6 months overall. The day of life at which the first saliva sample was collected had a mean of 2.4 days, with a standard deviation of 2.1 days. Subsequently, as previously described, samples were collected from each neonate every 7–10 days, until discharge from the NICU or until reaching 40 weeks of postmenstrual age, whichever occurred first. Overall, an average of eight samples per enrolled neonate was collected (with a standard deviation of five samples).

### 3.3. Baseline Comparison

Tβ4 was detected in 148 samples (99.3%) and Tβ10 in 143 samples (96%). Their oxidized forms were present, in varying proportions, in 141 (94.6%) and 131 (87.9%) samples, respectively.

The correlation between gestational age and the levels of both total Tβ4 and total Tβ10 and the percentage of their oxidized derivatives was not significant. In contrast, a significant negative correlation was observed between PMA and total Tβ4 (Spearman’s Rho −0.269, *p* = 0.001), as well as the percentage of oxidized Tβ4 (−0.184, *p* = 0.025), and between PMA and total Tβ10 (−0.166, *p* = 0.043).

Moreover, correlation analysis between Fraction of Inspired Oxygen (FiO_2_) (at the time of sample collection) and total levels of either of the proteins, and between FiO_2_ and the percentage of oxidized Tβ4 was not significant. Conversely, higher FiO_2_ values correlated with lower levels of oxidized Tβ10 (−0.230, *p* = 0.005) and with lower percentages of oxidized Tβ10 (−0.322, *p* < 0.001).

Considering the samples collected during the first week of life, there were no significant differences between neonates who subsequently developed BPD (any grade) and those who did not (Table 3).

### 3.4. Longitudinal Trajectories

To investigate temporal changes beyond the first week of life, a longitudinal analysis of Tβ10 and Tβ4 (total, oxidized, and percentage of oxidized forms) was performed across all available weekly samples, yielding the following results:

**Concerning Tβ10:**

- **Total Tβ10** (nl): the GEE model showed a significant interaction between BPD and week (Wald χ^2^ = 73.928; df = 2; *p* < 0.001). In neonates without BPD, total Tβ10 increased on average by 0.294 log units per week (95% CI: 0.115–0.474; *p* = 0.001), whereas in neonates with BPD, it slightly decreased by 0.125 log units per week (95% CI: −0.213 to −0.037; *p* = 0.006), indicating a divergent trend over time between the two groups;
- **Oxidized Tβ10** (nl): the GEE model showed a significant interaction between BPD and week (Wald χ^2^ = 159.435; *p* < 0.001). In neonates without BPD, oxidized Tβ10 increased on average by 0.526 log units per week (95% CI: 0.303–0.750; *p* < 0.001), whereas in neonates with BPD, the variation was not significant (−0.073 log units/week; 95% CI: −0.209 to 0.064; *p* = 0.296);
- **Percentage of oxidized Tβ10**: the BPD × Week interaction was highly significant (Wald χ^2^ = 112.609; df = 2; *p* < 0.001). In neonates without BPD, the percentage of oxidized thymosin increased on average by 2.80 units per week (95% CI: 2.21–3.39; *p* < 0.001), whereas in neonates with BPD, the increase was slower, 0.60 units per week (95% CI: 0.06–1.14; *p* = 0.030).

**Concerning Tβ4:**

- **Total Tβ4** (nl): the GEE model showed a significant interaction between BPD and week (Wald χ^2^ = 130.136; df = 2; *p* < 0.001). In neonates without BPD, total Tβ4 increased on average by 0.289 log units per week (95% CI: 0.091–0.487; *p* = 0.004), whereas in neonates with BPD, it slightly decreased by 0.133 log units per week (95% CI: −0.217 to −0.049; *p* = 0.002), indicating a divergent trend over time between the two groups;
- **Oxidized thymosin β4**: the longitudinal trend of log-transformed oxidized Tβ4 differed between groups (Wald χ^2^ = 78.900; df = 2; *p* < 0.001). In neonates without BPD, an increasing trend over time was observed (β = +0.247 log units/week; 95% CI: −0.006 to 0.500; *p* = 0.056), whereas in neonates with BPD, a significant reduction was observed (β = −0.161 log units/week; 95% CI: −0.307 to −0.014; *p* = 0.032);
- **Percentage of oxidized Tβ4**: no significant difference was observed in the temporal trend of the percentage of oxidized Tβ4 between groups (BPD × time interaction: Wald χ^2^ = 1.161; *p* = 0.560), suggesting that the relative oxidized fraction of the protein remains stable despite changes in absolute levels.

**Figure 3:**
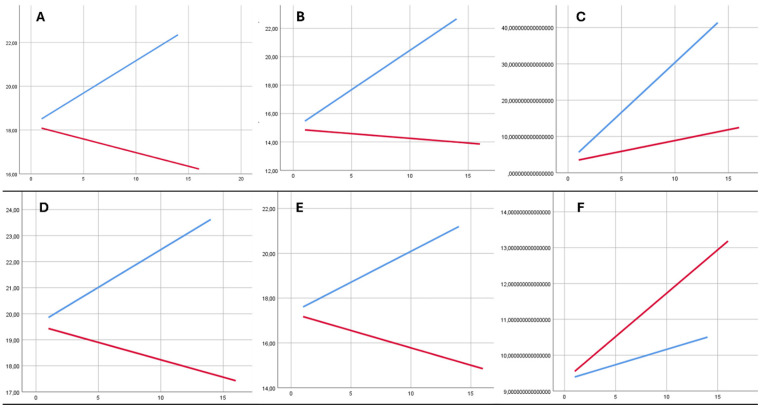
Longitudinal trends in salivary thymosin β4 and β10 levels over time. Blue line: Non-BPD group. Red line: BPD group. *Y*-axis: total Tβ10 (nl) (**A**); oxidized Tβ10 (nl) (**B**); percentage of oxidized Tβ10 (**C**); total Tβ4 (nl) (**D**); oxidized Tβ4 (nl) (**E**); percentage of oxidized Tβ4 (**F**). *X*-axis: weeks of life.

Figure 3 shows a graphical representation of the longitudinal trends of Tβ4 and Tβ10 (total, oxidized and percentage of oxidized form).

### 3.5. Clinical Correlation Analyses

Exploratory correlation analyses showed significant inverse associations between thymosin levels and cumulative respiratory support. Tβ4 was negatively correlated with duration of mechanical ventilation (Spearman’s Rho = −0.228, *p* = 0.025) and oxygen therapy (−0.336, *p* = 0.001). Oxidized Tβ4 was also negatively correlated with oxygen duration (−0.369, *p* < 0.001). Tβ10 showed a weak negative correlation with oxygen duration (−0.231, *p* = 0.024), while oxidized Tβ10 was negatively correlated with both duration of mechanical ventilation (−0.224, *p* = 0.028) and oxygen therapy (−0.341, *p* = 0.001).

## 4. Discussion

To our knowledge, this is the first study conducted to analyze the levels of Tβ4, Tβ10, and their oxidized derivatives in the oral fluid of preterm neonates, with the aim of exploring their potential association with BPD, a clinical condition related to oxidative stress.

The analysis confirmed a high presence of both total and oxidized forms of Tβ4 and Tβ10 in neonatal saliva, with detection rates exceeding 90% for both proteins. This finding supports the use of saliva as a reliable and non-invasive biological fluid for neonatal proteomic studies, especially for the detection of post-translationally modified proteins such as oxidized isoforms [48].

A key observation was the significant inverse correlation in salivary samples of the whole populations between PMA and the salivary levels of both total and oxidized Tβ4, and to a lesser extent, Tβ10. This indicates a progressive decline of these peptides as preterm infants mature. This finding aligns with the physiological trajectory described by Nemolato et al., who reported that Tβ4 levels rise during the very early neonatal period but subsequently decrease over time [49]. Our data, collected in extremely preterm infants over a longitudinal course, appear to capture this latter phase of physiological downregulation associated with extrauterine maturation. The lack of correlation with gestational age at birth further suggests that thymosin profiles are driven more by post-natal maturation and extrauterine environmental factors than by the degree of prematurity itself.

The development of BPD is a multi-factorial process, heavily influenced by postnatal complications. Our data confirm that the infants who developed BPD experienced a significantly higher burden of postnatal morbidities, including severe IVH (>grade 2), culture-proven sepsis, and pneumonia. These conditions are known to exacerbate systemic inflammation and oxidative stress. The significant difference in the incidence of IVH and sepsis between the two groups highlights the complex interplay between neurological, infectious, and pulmonary insults in the pathogenesis of BPD. The high incidence of sepsis and pneumonia in the BPD group underscores the role of infection-driven inflammation in driving the oxidative stress observed in our study. As expected, infants who developed BPD also required more intensive and prolonged respiratory support. All of these factors are well-known contributors to BPD development and reflect the greater clinical severity of this population. Accordingly, these differences should be considered when interpreting the results, as thymosin trajectories evolved differently over time in parallel with the clinical course of the two groups. Indeed, when comparing neonates who developed BPD to those who did not, no significant differences were observed in the first week of life for any parameter, including the percentage of oxidized Tβ10 (Table 2). However, longitudinal analysis revealed a divergent trend: in neonates without BPD, both thymosins showed a progressive increase over time in their total and, in part, oxidized forms, whereas in infants who developed BPD, this trend was blunted or reversed. Considering the clinical data, the divergent trends observed in the GEE models are particularly relevant. While the BPD group started with similar baseline levels in the first week, the subsequent weeks showed a progressive decline in oxidized Tβ4 and Tβ10 in the BPD group, coinciding with prolonged exposure to mechanical ventilation and oxygen. In contrast, the Non-BPD group, who required minimal respiratory support, showed a robust increase in these oxidized forms over time. Although still to be demonstrated, this may indicate that the inability to mount a progressive oxidative response (or the excessive consumption of these proteins) may be linked to the severity of the respiratory injury and the development of BPD.

In addition to the longitudinal analyses, exploratory correlation analyses were performed to assess the relationship between thymosin levels and cumulative respiratory support. These analyses showed weak-to-moderate inverse correlations between thymosin β4, thymosin β10 (and their oxidized forms), and duration of mechanical ventilation and oxygen therapy. Overall, higher respiratory support burden was associated with lower thymosin levels, suggesting a potential link between systemic illness severity and thymosin expression. However, the relatively modest strength of these associations suggests that respiratory support alone may not fully account for the observed longitudinal changes in thymosin trajectories.

Another important finding was the significant inverse correlation between FiO_2_ and both the absolute level and percentage of oxidized Tβ10. No such correlation was found for Tβ4. This selective relationship is of potential biological relevance, as FiO_2_ represents a direct proxy of oxygen exposure and, consequently, of exogenous oxidative stress burden in preterm neonates. The fact that only oxidized Tβ10—but not oxidized Tβ4—showed this association may indicate differential susceptibility of these two thymosin isoforms to oxygen-driven redox alterations, possibly reflecting distinct structural stability, turnover, or compartmentalization under hyperoxic conditions. From a pathophysiological perspective, this finding may suggest that Tβ10 is more dynamically responsive to changes in oxygen availability and/or more rapidly consumed/modified in the presence of increased oxidative stress. Alternatively, it may reflect a differential regulation of release or degradation pathways between the two peptides during neonatal adaptation to respiratory support. Although these interpretations remain speculative, the observed isoform-specific behavior further supports the concept that Tβ4 and Tβ10 may not be functionally redundant in the context of neonatal lung disease and oxidative stress. This differential pattern warrants further mechanistic investigation in larger and more homogeneous cohorts.

The interpretation of all of these results must necessarily consider the exploratory nature of this study. The intrinsic differences between the two groups of neonates studied—particularly the significantly lower gestational age and birth weight in the BPD group, as well as the significantly longer duration of mechanical ventilation, non-invasive respiratory support, and oxygen therapy in these infants, together with the higher incidence of sepsis and pneumonia episodes—are all factors that, per se, justify the development of BPD. As this was a pilot study not designed to assess antioxidant capacity or inflammation, we can only state that the observed patterns may reflect differences in oxidative stress exposure and clinical severity. Conversely, current evidence regarding Tβ4 suggests that it exerts anti-inflammatory, cytoprotective, and tissue-repair-promoting effects, including modulation of cell migration, angiogenesis, and epithelial regeneration. The observed reduction over time in total and oxidized thymosin β4 in infants with BPD may therefore reflect an insufficient reparative response to ongoing lung injury and inflammation, which are hallmarks of BPD pathophysiology. Considering the scientific evidence regarding the potential biological roles of oxidized Tβ4, no studies are currently available in the literature on the possible functions of oxidized Tβ10 [30,33,34,35,36,37,38,50,51]. However, in this study, thymosin β10 showed a divergent longitudinal pattern, with a progressive increase in infants without BPD and a decrease or lack of significant increase in those with BPD. Although this remains to be demonstrated, it may indicate an altered adaptive or redox response in infants prone to developing chronic lung disease.

Interestingly, while absolute concentrations differed in their trajectories, the percentage of oxidized Tβ4 did not show significant differences between groups, suggesting that the relative redox balance of this protein may remain stable despite changes in total levels. In contrast, the more pronounced divergence observed for oxidized Tβ10 and its percentage form may point to a potentially greater involvement of Tβ10 in oxidative stress-related mechanisms.

The study results provide several insights and encourage the design of further mechanistic and experimental studies with the aim of clarifying whether the observed alterations represent a contributory factor in BPD pathogenesis or an early biomarker of evolving lung injury. Only a larger study—allowing the analysis of thymosin trajectories among neonates with comparable gestational age and birth weight, and with postnatal courses not significantly differing in terms of exposure to ventilation, oxygen therapy, and infectious episodes—could clarify the potential role of Tβ4 and/or Tβ10 as disease biomarkers and possibly demonstrate that thymosin oxidation may represent not merely a detrimental modification but rather a functionally relevant post-translational modification capable of modulating cellular and inflammatory responses. One possible hypothesis is that Tβ4 and Tβ10 participate in postnatal lung adaptation and repair processes, and that altered longitudinal patterns of these peptides may be associated with the development of BPD.

This study has several limitations, as evident from the analysis of the results. First of all, the small sample size, which may have limited statistical power to detect more subtle differences or to conduct multivariate analysis.

Additionally, the heterogeneity of sample collection times and individual clinical variability may have influenced the salivary levels of the studied proteins.

Lastly, since this is an observational study, no causal link can be established between oxidized thymosin levels and the development of an oxidative stress-related disease such as BPD.

The present pilot study was not designed to identify disease biomarkers but rather to serve as an exploratory investigation of oxidized and non-oxidized Tβ10 and Tβ4 levels in infants who develop BPD compared with those who do not develop the disease.

Despite these limitations, our findings suggest that salivary analysis, particularly of oxidized Tβ10 forms, represents a promising tool for exploring oxidative status in preterm neonates and salivary thymosins emerge as promising candidates for investigation in future studies as early biomarkers to identify preterm neonates at risk of conditions like BPD.

At present, there is not enough evidence to consider these proteins as pathognomonic markers for disease. Further studies with larger cohorts and extended clinical follow-up will be needed to validate these findings and clarify the functional significance of the observed oxidative modifications. Larger studies could confirm the longitudinal trends in oxidized Tβ10 and Tβ4 levels and could allow the identification of a cut-off value for oxidized Tβ4 and Tβ10 levels at a specific postnatal time point in preterm infants, allowing this biomarker to be used as a predictor of subsequent BPD development.

Moreover, this study once again highlights how salivary proteomic analysis is a non-invasive approach that is well tolerated even by extremely preterm neonates. This finding is particularly relevant considering the extreme fragility of this patient population. These characteristics make it possible to collect serial samples, thereby enabling longitudinal monitoring. The method proved to be reliable for the identification and quantification of proteins and their post-translational modifications.

## 5. Conclusions

In conclusion, this pilot study demonstrates that salivary Tβ4, Tβ10, and their oxidized forms can be reliably detected in preterm neonates and supports the feasibility of saliva as a non-invasive matrix for longitudinal proteomic monitoring in this highly vulnerable population.

The distinct longitudinal trajectories observed between infants who developed BPD and those who did not—particularly for oxidized Tβ10—suggest a possible association between thymosin regulation, oxidative stress exposure, and postnatal lung development. However, given the exploratory nature of the study and the substantial differences in gestational age, birth weight, respiratory support, and infectious burden between groups, these findings should be interpreted with caution and cannot establish a causal relationship with BPD pathogenesis. Further studies in larger and clinically more homogeneous cohorts will be necessary to validate these findings, clarify the mechanistic role of oxidized thymosins in neonatal lung disease, and determine whether altered salivary thymosin profiles may serve as early indicators of infants at increased risk for BPD.

## Figures and Tables

**Table 1:** Data are reported as median [interquartile range] and number (percentage).

	General Characteristics
Gestational age, weeks	28.0 [25.7–29.6]
Neonatal weight, grams	940 [790–1180]
Male sex	11/18 (61.1%)
Vaginal delivery	6/18 (33.3%)
BPD	7/18 (38.9%)

**Table 2:** Data are reported as mean ± standard deviation, median [interquartile range] and number (percentage). Categorical data were compared using the Chi-square test or Fisher’s exact test. Continuous variables were compared using the Student’s *t*-test for normally distributed data or the Mann–Whitney U test for non-normally distributed data. IVH: Intraventricular Hemorrhage; ROP: Retinopathy of Prematurity; PDA: Patent Ductus Arteriosus. Statistically significant *p*-values are shown in bold.

	BPD Group (7)	Non-BPD Group (11)	*p*-Value
Gestational age (GA)	25.65 ± 1.54	28.95 ± 1.81	**0.001**
Birth weight	760 ± 100	1130 ± 264	**0.001**
Female sex	2 (28.6%)	5 (45.5%)	0.637
Vaginal delivery	2 (28.6%)	4 (36.4%)	1
Length of stay	133 [129–179]	75 [57–80]	**<0.001**
PROM (h)	82 [0–168]	0 [0–22]	0.151
PROM > 3 weeks	1 (14.3%)	0	0.389
Chorioamnionitis	1 (14.3%)	1 (9.1%)	1
Small for GA	1 (14.3%)	2 (18.2%)	1
Apgar 1′	7 [5–7]	6 [3–7]	0.724
Apgar 5′	8 [8–9]	8 [7–8]	0.211
Surfactant administration	6 (85.7%)	5 (45.5%)	0.151
Surfactant (number of doses)	2 [1–2]	0 [0–1]	0.085
Mechanical ventilation (h)	600 [456–1104]	0 [0–17]	**<0.001**
Non-invasive ventilation (days)	80 [47–113]	30 [10–34]	**0.001**
Oxygen (days)	72 [61–150]	0 [0–1]	**<0.001**
PDA hemodynamically significant	6 (85.7%)	3 (27.3%)	0.050
IVH > 2°	3 (42.9%)	0	**0.043**
ROP > 2°	3 (42.9%)	1 (9.1%)	0.245
Sepsis with positive hemoculture	1 [1–2]	0 [0–0]	**0.006**
Pneumoniae	2 [1–2]	0 [0–0]	**0.003**
Necrotizing enterocolitis	0	0	1

**Table 3:** Levels of total and oxidated proteins (natural logarithm) and percentage of oxidized forms obtained in the first week of life in the two groups. T-test for independent samples.

	BPD Group	Non-BPD Group	*p*-Value
Tβ_4_ (ln)	19.9 ± 2.9	21.3 ± 0.8	0.813
Tβ_10_ (ln)	18.0 ± 2.1	19.8 ± 0.8	0.279
Tβ_4_ ox(ln)	17.7 ± 4.3	18.3 ± 0.8	0.823
Tβ_10_ ox(ln)	14.5 ± 3.0	16.5 ± 0.2	0.367
%oxTβ_4_	14.5 ± 10.1	3.0 ± 2.5	0.355
%oxTβ_10_	4.7 ± 2.4	3.2 ± 3.0	0.690

## Data Availability

The data that support the findings of this study are available upon request from the corresponding author due to privacy reasons.

## Abbreviations

The following abbreviations are used in this manuscript:

ROS	Reactive Oxygen Species
NICU	Neonatal Intensive Care Unit
BPD	Bronchopulmonary dysplasia
IVH	Intraventricular hemorrhage
PVL	Periventricular leukomalacia
NEC	Necrotizing enterocolitis
VEGF	Vascular endothelial growth factor
HIF	Hypoxia-induced transcription factor
BALF	Bronchoalveolar lavage fluid
Tβ4	Thymosin β4
Tβ10	Thymosin β10
PTM	Post-translational modification
Tβ4-SO	Tβ4-sulfoxide
PMA	Post-Menstrual Age
GA	Gestational age
FiO_2_	Fraction of Inspired Oxygen
RP–HPLC-ESI-MS	Reversed-Phase High-Performance Liquid Chromatography coupled with Electrospray Ionization Mass Spectrometry

## References

[1] Ozsurekci Y., Aykac K. (2016). Oxidative Stress Related Diseases in Newborns. Oxidative Med. Cell. Longev..

[2] Poljšak B., Fink R. (2014). The protective role of antioxidants in the defence against ROS/RNS-mediated environmental pollution. Oxidative Med. Cell. Longev..

[3] Jomova K., Raptova R., Alomar S.Y., Alwasel S.H., Nepovimova E., Kuca K., Valko M. (2023). Reactive oxygen species, toxicity, oxidative stress, and antioxidants: Chronic diseases and aging. Arch. Toxicol..

[4] Torres-Cuevas I., Parra-Llorca A., Sánchez-Illana A., Nuñez-Ramiro A., Kuligowski J., Cháfer-Pericás C., Cernada M., Escobar J., Vento M. (2017). Oxygen and oxidative stress in the perinatal period. Redox Biol..

[5] Moore T.A., Ahmad I.M., Zimmerman M.C. (2018). Oxidative Stress and Preterm Birth: An Integrative Review. Biol. Res. Nurs..

[6] La Verde M., De Franciscis P., Molitierno R., Caniglia F.M., Fordellone M., Braca E., Carbone C., Varro C., Cirillo P., Scappaticcio L. (2025). Thyroid Hormones in Early Pregnancy and Birth Weight: A Retrospective Study. Biomedicines.

[7] Zhou B., Chen Y., Cai W., Liu L., Hu X. (2020). Effect of Gestational Weight Gain on Associations Between Maternal Thyroid Hormones and Birth Outcomes. Front. Endocrinol..

[8] Maraka S., Ospina N.M.S., O’Keeffe D.T., Espinosa De Ycaza A.E., Gionfriddo M.R., Erwin P.J., Coddington C.C., Stan M.N., Murad M.H., Montori V.M. (2016). Subclinical hypothyroidism in pregnancy: A systematic review and meta-analysis. Thyroid.

[9] Perez M., Robbins M.E., Revhaug C., Saugstad O.D. (2019). Oxygen radical disease in the newborn, revisited: Oxidative stress and disease in the newborn period. Free Radic. Biol. Med..

[10] Cannavò L., Perrone S., Viola V., Marseglia L., Di Rosa G., Gitto E. (2021). Oxidative Stress and Respiratory Diseases in Preterm Newborns. Int. J. Mol. Sci..

[11] Peña-Bautista C., Durand T., Vigor C., Oger C., Galano J.M., Cháfer-Pericás C. (2019). Non-invasive assessment of oxidative stress in preterm infants. Free Radic. Biol. Med..

[12] De Almeida V.O., Pereira R.A., Amantéa S.L., Rhoden C.R., Colvero M.O. (2022). Neonatal diseases and oxidative stress in premature infants: An integrative review. J. Pediatr..

[13] Gerstner B., DeSilva T.M., Genz K., Armstrong A., Brehmer F., Neve R.L., Felderhoff-Mueser U., Volpe J.J., Rosenberg P.A. (2008). Hyperoxia causes maturation-dependent cell death in the developing white matter. J. Neurosci..

[14] Volpe J. (2001). Neurobiology of Periventricular Leukomalacia in the Premature Infant. Pediatr. Res..

[15] Marseglia L., D’Angelo G., Manti S., Aversa S., Reiter R.J., Antonuccio P., Centorrino A., Romeo C., Impellizzeri P., Gitto E. (2015). Oxidative stress mediated damage in newborns with necrotizing enterocolitis: A possible role of melatonin. Am. J. Perinatol..

[16] Perrone S., Tataranno M.L., Santacroce A., Negro S., Buonocore G. (2014). The role of oxidative stress on necrotizing enterocolitis in very low birth weight infants. Curr. Pediatr. Rev..

[17] Kimble A., Robbins M.E., Perez M. (2022). Pathogenesis of Bronchopulmonary Dysplasia: Role of Oxidative Stress from ‘Omics’ Studies. Antioxidants.

[18] Wang J., Dong W. (2018). Oxidative stress and bronchopulmonary dysplasia. Gene.

[19] Ferrante G., Montante C., Notarbartolo V., Giuffrè M. (2022). Antioxidants: Role the in prevention and treatment of bronchopulmonary dysplasia. Paediatr. Respir. Rev..

[20] Capasso L., Vento G., Loddo C., Tirone C., Iavarone F., Raimondi F., Dani C., Fanos V. (2019). Oxidative Stress and Bronchopulmonary Dysplasia: Evidences From Microbiomics, Metabolomics, and Proteomics. Front. Pediatr..

[21] Tirone C., Iavarone F., Tana M., Lio A., Aurilia C., Costa S., Castagnola M., Messana I., Vento G. (2021). Oxidative and Proteolytic Inactivation of Alpha-1 Antitrypsin in Bronchopulmonary Dysplasia Pathogenesis: A Top-Down Proteomic Bronchoalveolar Lavage Fluid Analysis. Front. Pediatr..

[22] Gitto E., Pellegrino S., Gitto P., Barberi I., Reiter R.J. (2009). Oxidative stress of the newborn in the pre- and postnatal period and the clinical utility of melatonin. J. Pineal Res..

[23] Varsila E., Pesonen E., Andersson S. (1995). Early protein oxidation in the neonatal lung is related to development of chronic lung disease. Acta Paediatr..

[24] Vento G., Mele M.C., Mordente A., Romagnoli C., Matassa P.G., Zecca E., Zappacosta B., Persichilli S. (2000). High total antioxidant activity and uric acid in tracheobronchial aspirate fluid of preterm infants during oxidative stress: An adaptive response to hyperoxia?. Acta Paediatr..

[25] Ogihara T., Hirnao K., Morinobu T., Kim H.-S., Hiroi M., Ogihara H., Tami H. (1999). Raised concentrations of aldehyde lipid peroxidation products in premature infants with chronic lung disease. Arch. Dis. Child. Fetal Neonatal Ed..

[26] Inder T.E., Graham P., Sanderson K., Taylor B.J. (1994). Lipid peroxidation as a measure of oxygen free radical damage in the very low birthweight infant. Arch. Dis. Child. Fetal Neonatal Ed..

[27] Gladstone I.M., Levine R.L. (1994). Oxidation of proteins in neonatal lungs. Pediatrics.

[28] Piersigilli F., Bhandari V. (2016). Biomarkers in neonatology: The new “omics” of bronchopulmonary dysplasia. J. Matern. Fetal Neonatal Med..

[29] Liu C.Q., Liu X.Y., Ouyang P.W., Liu Q., Huang X.M., Xiao F., Cui Y.H., Zhou Q., Pan H.W. (2023). Ferrostatin-1 attenuates pathological angiogenesis in oxygen-induced retinopathy via inhibition of ferroptosis. Exp. Eye Res..

[30] Kuzan A. (2016). Thymosin β as an Actin-binding Protein with a Variety of Functions. Adv. Clin. Exp. Med..

[31] Faa G., Messana I., Coni P., Piras M., Pichiri G., Piludu M., Iavarone F., Desiderio C., Vento G., Tirone C. (2024). Thymosin β_4_ and β_10_ Expression in Human Organs during Development: A Review. Cells.

[32] Hannappel E., Huff T. (2003). The thymosins. Prothymosin alpha, parathymosin, and beta-tymosins: Structure and function. Vitam. Horm..

[33] Smart N., Rossdeutsch A., Riley P.R. (2007). Thymosin beta4 and angiogenesis: Modes of action and therapeutic potential. Angiogenesis.

[34] Evans M.A., Smart N., Dubé K.N., Bollini S., Clark J.E., Evans H.G., Taams L.S., Richardson R., Lévesque M., Martin P. (2013). Thymosin b4-sulfoxide attenuates inflammatory cell infiltration and promotes cardiac wound healing. Nat. Commun..

[35] Young J.D., Lawrence A.J., MacLean A.G., Leung B.P., McInnes I.B., Canas B., Pappin D.J., Stevenson R.D. (1999). Thymosin beta 4 sulfoxide is an anti-inflammatory agent generated by monocytes in the presence of glucocorticoids. Nat. Med..

[36] Huff T., Zerzawy D., Hannappel E. (1995). Interactions of β-thymosins, thymosin β_4_-sulfoxide, and N-terminally truncated thymosin β_4_ with actin studied by equilibrium centrifugation, chemical cross-linking and viscometry. Eur. J. Biochem..

[37] Renga G., Oikonomou V., Stincardini C., Pariano M., Borghi M., Costantini C., Bartoli A., Garaci E., Goldstein A.L., Romani L. (2018). Thymosin β4 limits inflammation through autophagy. Expert Opin. Biol. Ther..

[38] Vasilopoulou E., Winyard P.J., Riley P.R., Long D.A. (2015). The role of thymosin-β4 in kidney disease. Expert Opin. Biol. Ther..

[39] Fanni D., Gerosa C., Nemolato S., Locci A., Marinelli V., Cabras T., Messana I., Fanos V., Castagnola M., Faa G. (2011). Thymosin beta 10 expression in developing human salivary glands. Early Hum. Dev..

[40] Zhang X., Ren D., Guo L., Wang L., Wu S., Lin C., Ye L., Zhu J., Li J., Song L. (2017). Thymosin beta 10 is a key regulator of tumorigenesis and metastasis and a novel serum marker in breast cancer. Breast Cancer Res..

[41] Li Z., Li Y., Tian Y., Li N., Shen L., Zhao Y. (2023). Pan-cancer analysis identifies the correlations of Thymosin Beta 10 with predicting prognosis and immunotherapy response. Front. Immunol..

[42] Castagnola M., Inzitari R., Fanali C., Iavarone F., Vitali A., Desiderio C., Vento G., Tirone C., Romagnoli C., Cabras T. (2011). The surprising composition of the salivary proteome of preterm human newborn. Mol. Cell. Proteom..

[43] Julious S.A. (2005). Sample size of 12 per group rule of thumb for a pilot study. Pharm. Stat..

[44] Zhang Z., Marshall G.A. (1998). A universal algorithm for fast and automated charge state deconvolution of electrospray mass-to-charge ratio spectra. J. Am. Soc. Mass Spectrom..

[45] Levin Y., Schwarz E., Wang L., Leweke F.M., Bahn S. (2007). Labelfree LC-MS/MS quantitative proteomics for large-scale biomarker discovery in complex samples. J. Sep. Sci..

[46] Ong S.E., Mann M. (2005). Mass spectrometry-based proteomics turns quantitative. Nat. Chem. Biol..

[47] Jensen E.A., Dysart K., Gantz M.G., McDonald S., Bamat N.A., Keszler M., Kirpalani H., Laughon M.M., Poindexter B.B., Duncan A.F. (2019). The Diagnosis of Bronchopulmonary Dysplasia in Very Preterm Infants. An Evidence-based Approach. Am. J. Respir. Crit. Care Med..

[48] Messana I., Manconi B., Cabras T., Boroumand M., Sanna M.T., Iavarone F., Olianas A., Desiderio C., Rossetti D.V., Vincenzoni F. (2023). The Post-Translational Modifications of Human Salivary Peptides and Proteins Evidenced by Top-Down Platforms. Int. J. Mol. Sci..

[49] Nemolato S., Messana I., Cabras T., Manconi B., Inzitari R., Fanali C., Vento G., Tirone C., Romagnoli C., Riva A. (2009). Thymosin beta(4) and beta(10) levels in pre-term newborn oral cavity and foetal salivary glands evidence a switch of secretion during foetal development. PLoS ONE.

[50] Young J.D., Gracie J.A., Stevenson R.D., Lawrence A.J., Liew F.Y., McInnes I.B. (2001). Thymosin beta4 sylphoxide: Potential role in resolution of inflammation?. Arthritis Res..

[51] Faa G., Piras M., Mancuso L., Coni P., Pichiri G., Orrù G., Fanni D., Gerosa C., Cao G., Taibi R. (2021). Thymosin beta-4 prenatal administration improves fetal development and halts side effects due to preterm delivery. Eur. Rev. Med. Pharmacol. Sci..

